# The association between triglyceride-glucose index, cardio-cerebrovascular diseases, and death in Korean adults: A retrospective study based on the NHIS-HEALS cohort

**DOI:** 10.1371/journal.pone.0259212

**Published:** 2021-11-04

**Authors:** Joungyoun Kim, Sang-Jun Shin, Hee-Taik Kang

**Affiliations:** 1 Mo-Im Kim Nursing Research Institute, Yonsei University, College of Nursing, Seoul, Republic of Korea; 2 Department of Information & Statistics, Chungbuk National University, Cheongju, Republic of Korea; 3 Department of Family Medicine, Chungbuk National University Hospital, Cheongju, Republic of Korea; 4 Department of Family Medicine, Chungbuk National University College of Medicine, Cheongju, Republic of Korea; Ospedale del Cuore G Pasquinucci Fondazione Toscana Gabriele Monasterio di Massa, ITALY

## Abstract

**Background:**

The triglyceride-glucose (TyG) index is a reliable indicator of insulin resistance. We aimed to investigate the TyG index in relation to cardio-cerebrovascular diseases (CCVDs and mortality.

**Methods:**

This retrospective study included 114,603 subjects. The TyG index was categorized into four quartiles by sex: Q_1_, <8.249 and <8.063; Q_2_, 8.249‒<8.614 and 8.063‒<8.403; Q_3_, 8.614‒< 8.998 and 8.403‒<8.752; and Q_4_, ≥8.998 and ≥8.752, in men and women, respectively. To calculate hazard ratios (HRs) and 95% confidence intervals (CIs) for the primary outcomes (CCVDs and all-cause mortality) and secondary outcomes (cardiovascular diseases [CVDs], cerebrovascular diseases [CbVDs], CCVD-related deaths, or all-cause deaths), Cox proportional hazards regression models were adopted.

**Results:**

Compared to Q_1_, the HRs (95% CIs) for the primary outcomes of Q_2_, Q_3_, and Q_4_ were 1.062 (0.981‒1.150), 1.110 (1.024−1.204), and 1.151 (1.058−1.252) in men and 1.099 (0.986−1.226), 1.046 (0.938−1.166), and 1.063 (0.954−1.184) in women, respectively, after adjusted for age, smoking status, drinking status, physical activity, body mass index, systolic blood pressure, low-density lipoprotein cholesterol, economic status, and anti-hypertensive medications. Fully adjusted HRs (95% CIs) for CVDs of Q_2_, Q_3_, and Q_4_ were 1.114 (0.969−1.282), 1.185 (1.031−1.363), and 1.232 (1.068−1.422) in men and 1.238 (1.017−1.508), 1.183 (0.971−1.440), and 1.238 (1.018−1.505) in women, respectively. The adjusted HRs (95% CIs) for ischemic CbVDs of Q_2_, Q_3_, and Q_4_ were 1.005 (0.850−1.187), 1.225 (1.041−1.441), and 1.232 (1.039−1.460) in men and 1.040 (0.821−1.316), 1.226 (0.981−1.532), and 1.312 (1.054−1.634) in women, respectively, while the TyG index was negatively associated with hemorrhagic CbVDs in women but not in men. The TyG index was not significantly associated with CCVD-related death or all-cause death in either sex.

**Conclusions:**

Elevated TyG index was positively associated with the primary outcomes (CCVDs and all-cause mortality) in men and predicted higher risk of CVDs and ischemic CbVDs in both sexes.

## Introduction

Cardiovascular diseases (CVDs) and cerebrovascular diseases (CbVDs), which include coronary heart disease, peripheral arterial disease, and deep vein thrombosis in addition to other diseases of the heart and brain, are the leading cause of death worldwide [1]. CVDs and CbVDs are the second and fourth most common causes of death in Korea, respectively [2]. These cardio-cerebrovascular diseases (CCVDs) are a large burden on public health and a considerable number can be avoided by early detection and active management of their risk factors such as a healthy lifestyle.

Insulin functions as an important hormone to stimulate glucose metabolism and lipogenesis and suppress lipid utilization. Insulin resistance means that insulin does not function properly in the target tissue. Thus, insulin resistance plays an important role in the development of diabetes mellitus and may be closely associated with atherosclerotic CCVDs, malignant neoplasms, and mortality [3–5]. The early detection of insulin resistance is important in reducing the burden of chronic diseases. The hyperinsulinemic-euglycemic glucose clamp method is the gold standard to measure insulin resistance [6]. However, it is difficult to apply this technique to a real-world clinical setting because of its complex measurement procedure. The homeostasis model assessment of insulin resistance (HOMA-IR) is frequently used in the clinical setting but also has limited applicability because insulin levels must be measured [7]. The triglyceride-glucose (TyG) index is a novel reliable indicator to detect the early phase of insulin resistance and type 2 diabetes [8–10]. The TyG index has the advantage of having a normal distribution but the disadvantage of being easily affected by ethnic group, dietary pattern, and alcohol intake [11–13]. Several studies have reported that the TyG index was superior to HOMA-IR in predicting type 2 diabetes in the Korean population [10]. In part, the dietary pattern of the Korean populations who consume high levels of carbohydrates and low levels of fat may contribute to the higher predictability of the TyG index.

This study aimed to examine whether the TyG index was associated with primary outcomes (CCVDs and all-cause mortality) in Korean adults, based on the Korean National Health Insurance Service (NHIS)-National Health Screening (HEALS) cohort. In addition, we further investigated the association between the TyG index and each secondary outcome after stratifying the primary outcomes into CVDs, CbVDs, CCVD-related death, and all-cause death.

## Materials and methods

### Study population

The NHIS-HEALS cohort included 514,794 subjects who were randomly selected from the 5.1 million examinees of the national health check-up program from January 2002 to December 2003. The age of the subjects was between 40 and 79 years at the end of December 2002. The cohort contains the lifestyle information of subjects and past medical history of diseases based on the self-reported questionnaires, diagnostic codes, and prescription information based on national insurance claim data, death information such as the main cause and date, and laboratory information from biennial national health check-up programs. The NHIS-HEALS cohort did not collect specific lipid profile such as triglycerides (TG) except for total cholesterol levels between 2002 and 2008. Thus, we should set 2009–2010 as the baseline because TG have been available since 2009.

**Fig 1:**
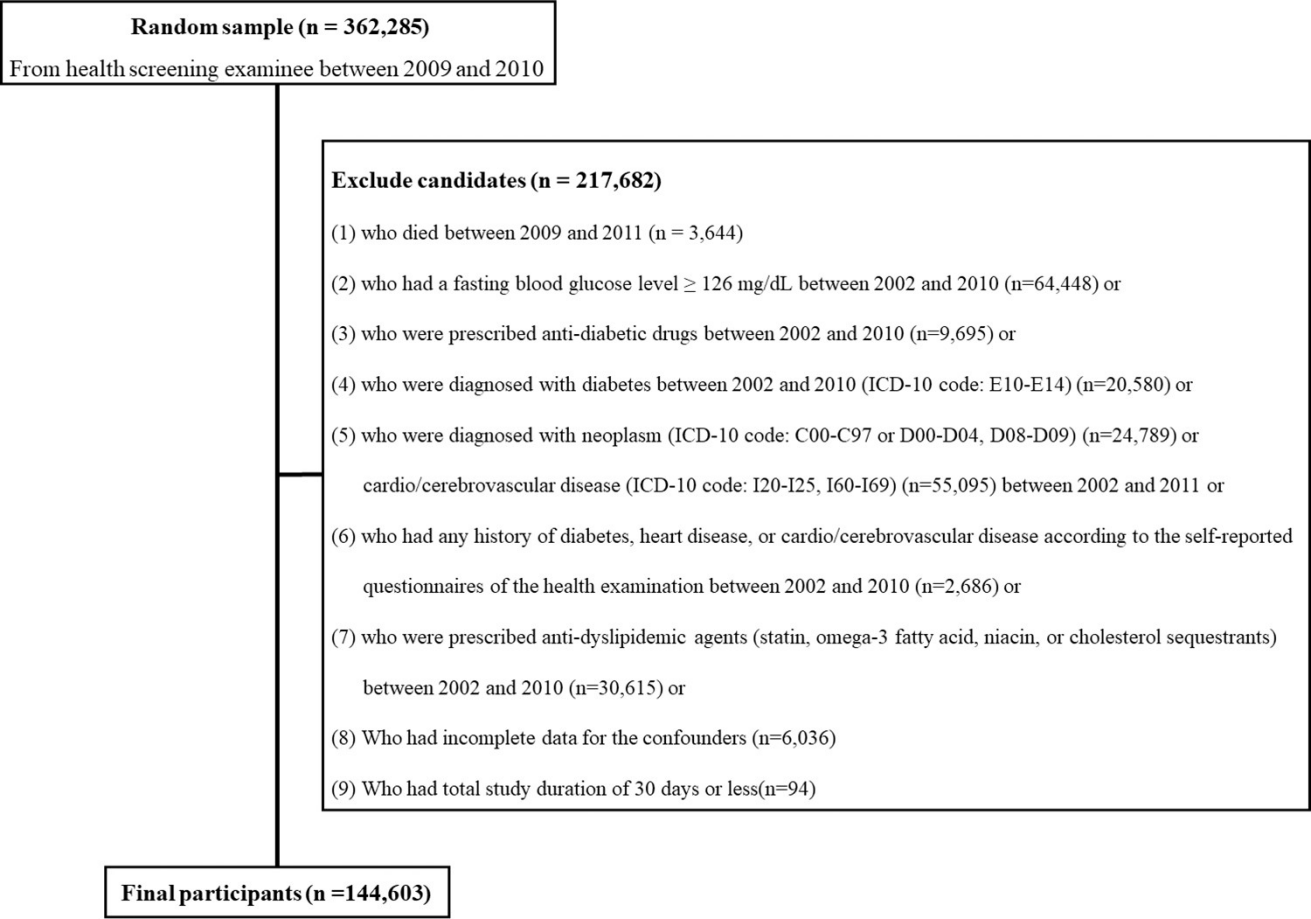
Flowchart of inclusion and exclusion criteria.

The flowchart describes the inclusions and exclusions of this study (Fig 1). Initially, 362,285 subjects who underwent the national health check-up programs between 2009 and 2010 were included. Among the initial subjects, individuals who met one or more of the following criteria were excluded: 1) subjects who died between 2009 and 2011 (n = 3644); 2) subjects who had a fasting blood glucose levels ≥ 126 mg/dL between 2002 and 2010 (n = 64,448); 3) subjects who were prescribed anti-diabetic drugs between 2002 and 2010 (n = 9695); 4) subjects who were diagnosed with diabetes between 2002 and 2010 (ICD-10 code: E10-E14) (n = 20,580); 5) subjects who were diagnosed with any neoplasm (ICD-10 code: C00-C97 or D00-D09) (n = 24,789) or CCVDs (the International Classification of Diseases [ICD]-10 code: I20-I25 for CVDs or I60-I69 for CbVDs) (n = 55,095) between 2002 and 2011; 6) subjects who had any history of diabetes, heart disease, CVDs, or CbVDs according to the self-reported questionnaires of the national health check-up program between 2002 and 2010 (n = 2,686); 7) subjects who were prescribed lipid-lowering agents (such as statin, omega-3 fatty acids, niacin, or cholesterol sequestrants) between 2002 and 2010 (n = 30,615); 8) subjects who had incomplete data for the confounders (n = 6036); or 9) subjects whose study duration was 30 days or less (n = 94). After these exclusions, 144,603 subjects (78,021 men and 66,582 women) were included in this study.

This research followed the 1964 Helsinki Declaration and was approved by the Institutional Review Board of Chungbuk National University Hospital (CBNUH 2021-03-003)

### Definition of TyG index, cardio-cerebrovascular disease, death, and study duration

The TyG index was calculated using the following equation: TyG = Ln (TG [mg/dL] × glucose [mg/dL]/2) [14, 15]. Calculated TyG indices were divided into four groups by sex: Q_1_, <8.249 in men and <8.063 in women; Q_2_, 8.249 ‒ <8.614 in men and 8.063 ‒ <8.403 in women; Q_3_, 8.614 ‒ < 8.998 in men and 8.403 ‒ <8.752 in women; and Q_4_, ≥8.998 in men and ≥8.752 in women.

The primary end point of this study is to compare the occurrence rates of CCVDs and all-cause mortality by TyG quartile groups after enrollment (2009‒2010). CCVDs included CVDs (I20-I25) and CbVDs (I60-I69) based on the ICD-10 code. Furthermore, CbVDs were further stratified in ischemic, hemorrhagic, and other CbVDs according to diagnostic code as follows: ischemic CbVDs were coded as I63 (cerebral infarction), I65 (occlusion and stenosis of precerebral arteries, not resulting in cerebral infarction), and I66 (occlusion and stenosis of cerebral arteries, not resulting in cerebral infarction); hemorrhagic CbVDs as I60 (subarachnoid hemorrhage), I61 (intracranial hemorrhage), and I62 (other nontraumatic intracranial hemorrhage); and other CbVDs as I64 (stroke, not specified as hemorrhage or infarction), I67 (other cerebrovascular diseases), I68 (cerebrovascular disorders in diseases classified elsewhere), and I69 (sequelae of cerebrovascular disease). CCVDs were defined when the main diagnosis (I20-I25 or I60-I69) was recorded at least twice in outpatients or once in hospitalized patients. As secondary outcomes, we considered the subgroup analyses for each CVD, CbVD, CCVD-related deaths, and all-cause deaths. In the case of deceased patients, it was classified by the cause of death recorded in the death certificate. If subjects died from fatal CCVD and the direct cause of death was recorded as CVD or CbVD on his/her death certificate, they were defined as CCVD-related deaths.

The research start date was defined as the day of the first health check-up at baseline (2009–2010). If a subject experienced CCVDs or death, the end date is the earlier date of the events. Otherwise, the end date is the last of the two following cases: the date of the last outpatient clinic visit or the last health check-up.

### Definition of covariates

Risk factors that can attribute to CCVDs or deaths were controlled such as age, body mass index (BMI), systolic blood pressure (SBP), low-density lipoprotein cholesterol (LDL-C), lifestyle (cigarette smoking, alcohol consumption, and physical activity), and economic status (monthly household income). These confounding factors were surveyed at baseline (2009‒2010).

BMI (unit, kg/m^2^) was calculated as body mass (kg) divided by squared height (m). Lifestyle and household income levels were collected from self-reported questionnaires. Smoking status was classified as ever smokers (who had smoked cigarettes in the past) and never smokers (who had never smoked cigarettes). Alcohol consumption was categorized into rare (less than once a week), sometimes (once to twice a week), and often (three or more times a week). Physical activity was divided into rare (less than once a week of any kind of exercise), sometimes (individuals who did not meet the definition of rare or regular physical activity), and regular (five or more times of walking or moderate-intensity exercise a week; three or more times of vigorous-intensity a week; four or more times of walking, moderate-intensity physical activity or vigorous-intensity exercise a week). Economic status was classified into three groups, based on the self-reported monthly household income: low, 0 to 30^th^ percentile; middle, 31^st^ to 70^th^ percentile; and high, 71^st^ to 100^th^ percentile.

### Statistical analysis

Continuous and categorical variables were presented as mean ± standard deviations (SDs) and the number of subjects (%), respectively. To check the group difference, analysis of variance (ANOVA) tests for continuous variables and chi-square tests and log-linear models for categorical variables were used. To investigate the association between the TyG index and outcomes (CVD, CbVD, and deaths), outcome-free survival rates were estimated and compared using the Kaplan-Meier method and the log-rank test. The hazard ratios (HRs) and 95% confidence intervals (CIs) were calculated to examine the association between the TyG index and the primary outcomes (CCVDs and all-cause mortality) based on Cox proportional hazards regression models after adjusting for confounders. Three Cox proportional hazards regression models were built after adjusting for age, smoking status, drinking status, physical activity, body mass index, systolic blood pressure, LDL-C, economic status, and anti-hypertensive medications. All p-values were two-sided and statistical significance was set as <0.05. The statistical analyses were performed using the statistical package SAS enterprise version 7.1 (SAS Inc., Cary, NC) and R studio version 3.3.3.

## Results

In total, 144,603 subjects (78,021 men and 66,582 women) were included and the median study duration was 5.97 years.

S1 Table displays the p-values for the simultaneous tests of effects by sex and TyG quartile on each variable. For the continuous variables, two-way ANOVA was employed. For the categorical variables, a log-linear model was used. For every variable, we found statistically significant effects of sex and TyG quartile as well as interactions. Based on this significant result, we stratified the data by sex.

Table 1 presents the baseline characteristics of the subjects according to the TyG quartile by sex. Mean age increased in men but decreased in women with the higher quartile group. In both sexes, BMI, SBP, glucose, TG, and percentage of ever smokers increased with increased quartile while the percentage of regular physical activity decreased (all p-values <0.001). Mean TyG index from Q_1_ to Q_4_ was 7.945, 8.438, 8.798, and 9.359, respectively, in men and 7.775, 8.240, 8.570, and 9.086, respectively, in women.

**Table 1:** Baseline characteristics according to the triglyceride-glucose index quartile.

Male	Q_1_ (<8.249)	Q_2_ (8.249‒<8.614)	Q_3_ (8.614‒<8.998)	Q_4_ (≥8.998)	p-valueX
Number (N)	19,534	19,481	19,523	19,483	N.A
Age, years	56.6 ± 8.1	56.2 ± 7.9	55.7 ± 7.6	54.7 ± 6.9	<0.001
BMI, kg/m^2^	22.7 ± 2.6	23.5 ± 2.6	24.1 ± 2.6	24.8 ± 2.6	<0.001
SBP, mmHg	121.8 ± 14.2	124.0 ± 14.2	125.6 ± 14.2	127.4 ± 14.2	<0.001
Glucose, mg/dL	89.9 ± 10.4	93.5 ± 10.6	96.0 ± 10.9	99.4 ± 11.2	<0.001
TG, mg/dL	65.1 ± 15.0	100.6 ± 15.2	140.4 ± 21.8	248.7 ± 98.3	<0.001
LDL-C, mg/dL	112.7 ± 31.6	118.8 ± 31.7	120.3 ± 32.6	109.8 ± 38.2	<0.001
TyG	7.945 ± 0.247	8.438 ± 0.105	8.798 ± 0.109	9.359 ± 0.310	<0.001
Ever smokers, N (%)	11,437 (58.5)	12,221 (62.7)	12,962 (66.4)	13,800 (70.8)	<0.001
Drinking status, N (%)					<0.001
Rare	7,623 (39.0)	7,262 (37.3)	6,681 (34.2)	5,708 (29.3)	
Sometimes	7,964 (40.8)	8,062 (41.4)	8,281 (42.4)	8,382 (43.0)	
Often	3,947 (20.2)	4,157 (21.3)	4,561 (23.4)	5,393 (27.7)	
Physical activity, N (%)					<0.001
Rare	3,954 (20.2)	4,069 (20.9)	4,068 (20.8)	4,208 (21.6)	
Sometimes	8,607 (44.1)	9,077 (46.6)	9,267 (47.5)	9,452 (48.5)	
Regular	6,973 (35.7)	6,335 (32.5)	6,188 (31.7)	5,823 (29.9)	
Economic status, N (%)					<0.001
Low	3,172 (16.2)	3,025 (15.5)	2,909 (14.9)	2,871 (14.7)	
Middle	5,890 (30.2)	5,994 (30.8)	5,742 (29.4)	5,924 (30.4)	
High	10,472 (53.6)	10,462 (53.7)	10,872 (55.7)	10,688 (54.9)	
Female	Q_1_ (<8.063)	Q_2_ (8.063‒<8.403)	Q_3_ (8.403‒<8.752)	Q_4_ (≥8.752)	p-value
Number (N)	16,666	16,645	16,625	16,646	N.A
Age, years	54.1 ± 6.7	55.6 ± 7.5	56.9 ± 8.2	58.3 ± 8.6	<0.001
BMI, kg/m^2^	22.6 ± 2.6	23.2 ± 2.7	23.6 ± 2.9	24.3 ± 2.9	<0.001
SBP, mmHg	117.5 ± 14.3	119.8 ± 14.7	122.3 ± 15.2	124.4 ± 15.3	<0.001
Glucose, mg/dL	87.6 ± 9.4	91.1 ± 9.5	93.1 ± 9.9	96.1 ± 10.6	<0.001
TG, mg/dL	56.1 ± 12.2	84.5 ± 11.8	115.0 ± 16.2	194.4 ± 73.8	<0.001
LDL-C, mg/dL	116.7 ± 30.9	122.6 ± 30.3	125.5 ± 33.7	122.5 ± 36.1	<0.001
TyG	7.775 ± 0.237	8.240 ± 0.097	8.570 ± 0.100	9.086 ± 0.290	<0.001
Ever smokers, N (%)	282 (1.7)	308 (1.9)	351 (2.1)	423 (2.5)	<0.001
Drinking status, N (%)					<0.001
Rare	13,545 (81.3)	13,871 (83.3)	14,087 (84.7)	14,233 (85.5)	
Sometimes	2,671 (16.0)	2,336 (14.0)	2,133 (12.8)	1,988 (11.9)	
Often	450 (2.7)	438 (2.6)	405 (2.4)	425 (2.6)	
Physical activity, N (%)					<0.001
Rare	4,334 (26.0)	4,715 (28.3)	4,941 (29.7)	5,181 (31.1)	
Sometimes	7,259 (43.6)	7,130 (42.8)	7,078 (42.6)	6,999 (42.0)	
Regular	5,073 (30.4)	4,800 (28.8)	4,606 (27.7)	4,466 (26.8)	
Economic status, N (%)					0.013
Low	4,373 (26.2)	4,291 (25.8)	4,236 (25.5)	4,262 (25.6)	
Middle	5,603 (33.6)	5,694 (34.2)	5,818 (35.0)	5,910 (35.5)	
High	6,690 (40.1)	6,660 (40.0)	6,571 (39.5)	6,474 (38.9)	

Abbreviations: BMI, body mass index; SBP, systolic blood pressure; TG, triglycerides; LDL-C, low-density lipoprotein cholesterol; TyG, triglycerides-glucose index.

p-values are calculated from one-way ANOVA in case of continuous variables and from chi square test in case of categorical variables.

**Fig 2:**
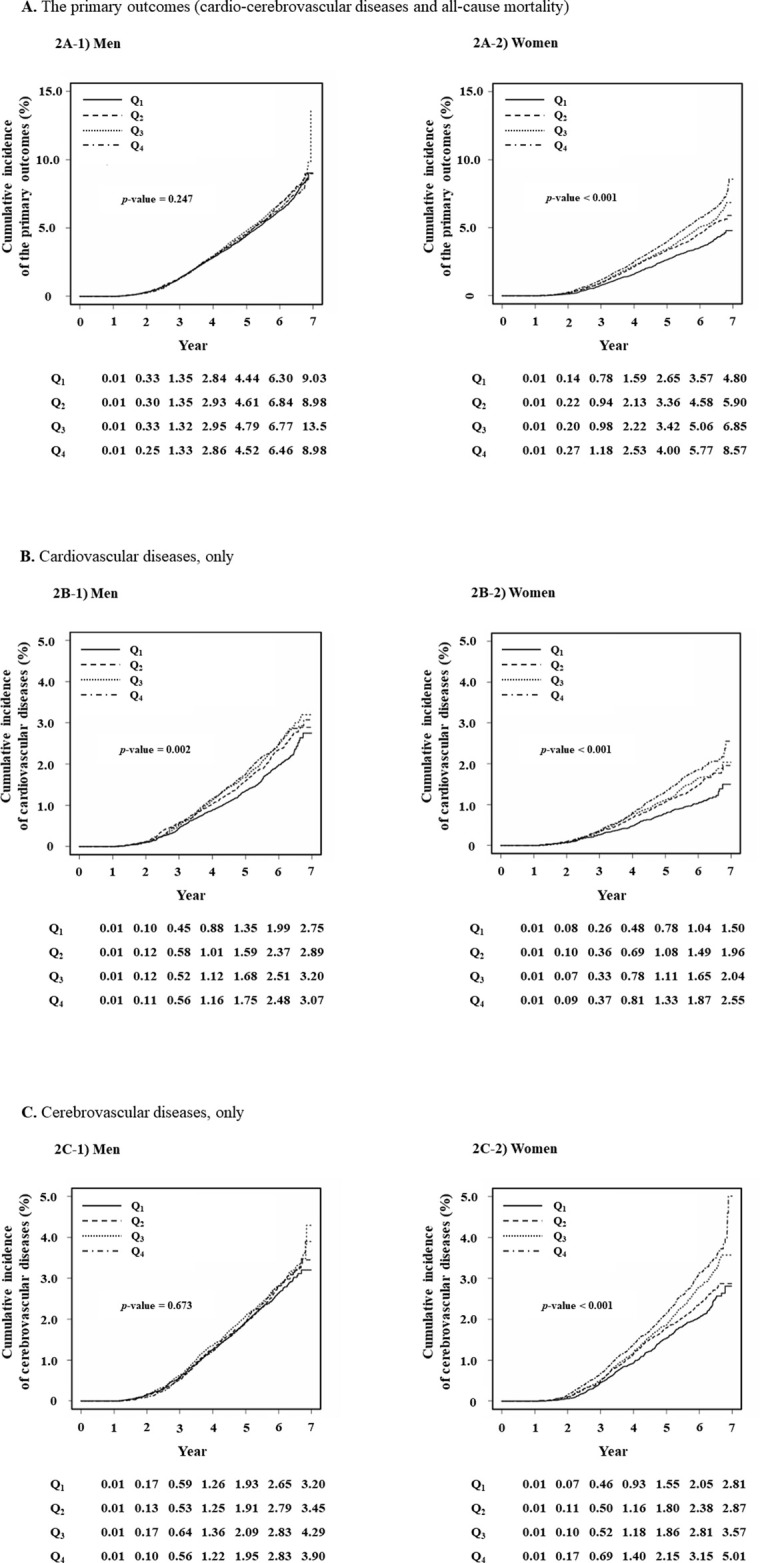
The estimated cumulative incidence of each outcome. A) The primary outcomes (cardio-cerebrovascular diseases and all-cause mortality). B) Cardiovascular diseases, only. C) Cerebrovascular diseases, only. All p-values are from log-rank tests.

Fig 2 shows the estimated cumulative event incidence, based on the Kaplan-Meier’s survival curve: 2A) the primary outcomes (CCVDs and all-cause mortality), 2B) CVDs, and 2C) CbVDs. Cumulative incidence of the primary outcomes was not different in men but significantly increased with the increased quartile groups in women (p-value = 0.247 in men and <0.001 in women) (Fig 2A). Further statistical analyses were conducted after dividing CCVDs into CVDs and CbVDs. The cumulative incidence of CVDs significantly increased with the increasing TyG index (all p-values <0.05) (Fig 2B). Cumulative incidence of CbVDs was not different in men (p-value = .0.673) but the highest in the Q_4_ of women (p-value <0.010) (Fig 2C). In addition, the Kaplan-Meier estimates for ischemic, hemorrhagic, and other CbVDs were plotted in S1 Fig.

Table 2 demonstrates the results of Cox proportional hazards regression models to examine the association between the TyG index and individual outcomes. Compared to Q_1_, HRs (95% CIs) for the primary outcomes of Q_2_, Q_3_, and Q_4_ were 1.062 (0.981‒1.150), 1.110 (1.024−1.204), and 1.151 (1.058−1.252), respectively, in men and 1.099 (0.986−1.226), 1.046 (0.938−1.166), and 1.063 (0.954−1.184), respectively, in women after adjusting for age, smoking status, alcohol consumption, physical activity, BMI, SBP, LDL-C, economic status, and anti-hypertensive medications. Subgroup analyses were performed to examine the association between the TyG index and individual outcomes (CVDs, CbVDs, CCVD-death, and all-cause death). Compared to Q_1_, HRs (95% CIs) for CVDs of Q_2_, Q_3_, and Q_4_ were 1.114 (0.969−1.282), 1.185 (1.031−1.363), and 1.232 (1.068−1.422), respectively, in men and 1.238 (1.017−1.508), 1.183 (0.971−1.440), and 1.238 (1.018−1.505), respectively, in women after fully adjusting. CbVDs were further classified into ischemic, hemorrhagic, and other CbVDs. The adjusted HRs (95% CIs) for ischemic CbVDs of Q_2_, Q_3_, and Q_4_ were 1.005 (0.850−1.187), 1.225 (1.041−1.441), and 1.232 (1.039−1.460) in men and 1.040 (0.821−1.316), 1.226 (0.981−1.532), and 1.312 (1.054−1.634) in women, respectively, while TyG index was negatively associated with hemorrhagic CbVDs in women but not in men. The adjusted HRs (95% CIs) for hemorrhagic CbVDs of Q_2_, Q_3_, and Q_4_ were 0.972 (0.642−1.472), 0.554 (0.347−0.884), and 0.452 (0.278−0.734) in women, compared to Q_1_. The TyG index was not significantly associated with CCVD-related death and all-cause death in both sexes.

**Table 2:** Cox-proportional hazards regression model for cardiovascular diseases, cerebrovascular diseases, cardio-cerebrovascular diseases, cardio-cerebrovascular diseases-related death, or all-cause death according to the TyG index quartile.

**Total**		**Men**	**Women**
The primary outcomes (Cardio-cerebrovascular diseases and all-cause mortality)	Q_1_	Reference	Reference
	Q_2_	1.062 (0.981−1.150)	1.099 (0.986−1.226)
	Q_3_	1.110 (1.024−1.204)	1.046 (0.938−1.166)
	Q_4_	1.151 (1.058−1.252)	1.063 (0.954−1.184)
**Subgroups**		**Men**	**Women**
Cardiovascular diseases	Q_1_	Reference	Reference
	Q_2_	1.114 (0.969−1.282)	1.238 (1.017−1.508)
	Q_3_	1.185 (1.031−1.363)	1.183 (0.971−1.440)
	Q_4_	1.232 (1.068−1.422)	1.238 (1.018−1.505)
Ischemic cerebrovascular diseases	Q_1_	Reference	Reference
	Q_2_	1.005 (0.850−1.187)	1.040 (0.821−1.316)
	Q_3_	1.225 (1.041−1.441)	1.226 (0.981−1.532)
	Q_4_	1.232 (1.039−1.460)	1.312 (1.054−1.634)
Hemorrhagic cerebrovascular diseases	Q_1_	Reference	Reference
	Q_2_	1.126 (0.781−1.625)	0.972 (0.642−1.472)
	Q_3_	0.937 (0.634−1.384)	0.554 (0.347−0.884)
	Q_4_	0.905 (0.602−1.360)	0.452 (0.278−0.734)
Other cerebrovascular diseases	Q_1_	Reference	Reference
	Q_2_	1.007 (0.833−1.218)	0.956 (0.789−1.160)
	Q_3_	0.949 (0.780−1.155)	0.943 (0.778−1.143)
	Q_4_	0.971 (0.793−1.189)	0.883 (0.727−1.073)
Cardio-cerebrovascular diseases-	Q_1_	Reference	Reference
related deaths	Q_2_	1.231 (0.778−1.947)	0.908 (0.405−2.035)
	Q_3_	1.157 (0.713−1.876)	1.452 (0.708−2.976)
	Q_4_	1.167 (0.698−1.954)	1.242 (0.601−2.566)
All-cause deaths	Q_1_	Reference	Reference
	Q_2_	1.048 (0.911−1.205)	1.216 (0.936−1.580)
	Q_3_	1.105 (0.955−1.278)	1.009 (0.776−1.313)
	Q_4_	1.063 (0.907−1.245)	0.990 (0.763−1.284)

Adjusted for age, smoking status (ever and never smokers), drinking status (rare, sometimes, and often) and physical activity (rare, sometimes, and regular), body mass index, systolic blood pressure, LDL-C, economic status (low, middle, and high), and anti-hypertensive medications.

Cerebrovascular diseases are as follows: ischemic cerebrovascular diseases, I63 (cerebral infarction), I65 (occlusion and stenosis of precerebral arteries, not resulting in cerebral infarction), and I66 (occlusion and stenosis of cerebral arteries, not resulting in cerebral infarction); hemorrhagic cerebrovascular diseases, I60 (subarachnoid hemorrhage), I61 (intracranial hemorrhage), and I62 (other nontraumatic intracranial hemorrhage); and other cerebrovascular diseases, I64 (stroke, not specified as hemorrhage or infarction), I67 (other cerebrovascular diseases), I68 (cerebrovascular disorders in diseases classified elsewhere), and I69 (sequelae of cerebrovascular disease).

## Discussion

This study demonstrated that there was a positive association between the TyG index primary outcomes (CCVDs or all-cause mortality) in men, but not in women. After stratifying the primary outcomes into CVDs, CbVDs, CCVD-related deaths, and all-cause deaths, a higher TyG index predicted a higher risk of CVDs and ischemic CbVDs in both sexes while it was inversely associated with hemorrhagic CbVDs in women. However, TyG index was not significantly associated with CCVD-related deaths and all-cause deaths in either sex.

Insulin stimulates glucose metabolism and suppresses fatty acid utilization and lipolysis as an energy source. Insulin resistance is defined as an impaired tissue response to insulin stimulation, resulting in the dysfunction of glucose and lipid metabolism [3]. These metabolic alterations lead to chronic hyperglycemia, dyslipidemia (in particular, increased free fatty acid release in adipose tissue and hepatic TG production), oxidative stress, and inflammation that induce endothelial dysfunction and atherosclerotic change. Eventually, insulin resistance increases CCVDs and premature death through these pathophysiologic mechanisms [3–5]. Thus, early detection and a more intensive management of insulin resistance are very important in reducing the likelihood of these events. Even though several methods to measure insulin resistance have been proposed, the hyperinsulinemic-euglycemic glucose clamp is considered the standard [6]. However, the hyperinsulinemic-euglycemic glucose clamp is not practical for use in real-world clinics because of complex and invasive measurements. Another reliable surrogate marker of insulin resistance is HOMA-IR. However, the disadvantage is that blood insulin levels are not routinely checked for patients without diabetes in the primary clinical setting [7].

The TyG index is newly proposed as a marker to measure insulin resistance. TG and glucose levels, which are used as variables to calculate the TyG index, are widely and commonly measured in clinics. The Korean Society of Lipid and Atherosclerosis recommends that the lipid profile (total cholesterol, TG, HDL-C, and LDL-C) be checked every 4‒6 years for adults to screen for dyslipidemia and CCVD risk [16]. Many guidelines recommend the annual measurement of fasting glucose levels to screen for diabetes or prediabetes [17, 18], while there are few recommendations for the measurement of fasting insulin levels to screen for them. Furthermore, Asian populations may consume more foods with a higher carbohydrate content than Western populations, which increases their blood TG and glucose levels [19]. Carbohydrate-rich diets raise the possibility of developing hypertriglyceridemia and impaired fasting glucose, which are related to the risk of CCVDs [20, 21]. Epidemiological studies support the proposal that the TyG index is a better indicator of insulin resistance in Asian populations than HOMA-IR [10, 22]. In addition, a lot of studies report that the TyG index was more closely associated with atherosclerosis, arterial stiffness, subclinical cerebral vessel diseases, and coronary arterial diseases than HOMA-IR [23–26].

The primary outcomes of this study are CCVDs and all-cause mortality. CCVDs are composed of CVDs (such as angina pectoris and myocardial infarctions) and CbVDs (such as non-traumatic cerebral hemorrhage, cerebral infarction, and cerebral arterial diseases). The statistical significance between the TyG index and primary outcomes differ by statistical method (significant only in women from the log-rank test and only in men from Cox proportional hazards model). The association was not significant from the log-rank test in men (Fig 2). However, it was significant after adjusting for age in the Cox proportional hazards regression model (S2 Table). This phenomenon is the opposite in women. That is, the log-rank test showed a significant association, but the fully adjusted Cox proportional hazards regression model showed non-significance. This might be explained by the different age distribution over TyG index by sex. As TyG quartile increases, the mean age decreases in men but increases in women. Because the log-rank test does not consider any confounding factors, the significance in women might include the compounded effect of age and TyG index. However, the elevated TyG index increased the occurrence of CVDs in both sexes, while it was not independently associated with individual events such as CbVDs, CCVD-related deaths, and all-cause deaths. These findings signify that the TyG index might be a good indicator in predicting CVDs such as ischemic heart diseases. Results from this study are consistent with previous studies by Park and Zhao et. al., who reported that an elevated TyG index forecast risk of ischemic heart disease and cardiovascular events [27, 28]. However, these studies had a smaller population size and did not stratify the study population according to sex. Our study supports the previous studies but clarifies the association between TyG index and individual events. There is lack of evidence to show the inverse association between TyG index and hemorrhagic stroke. Several studies have reported that higher TyG index was not significantly associated with intracranial hemorrhage but increased ischemic stroke [29, 30]. The inverse relationship of the TyG index to hemorrhagic CbVDs in this study is little different from this null association of previous studies. The protective mechanism through which the higher TyG index decreased the risk of hemorrhagic CbVDs should be further investigated.

This study has several strengths to distinguish it from previous studies. First, the NHIS-HEALS cohort represents the entire Korean adult population. The NHIS provides reliable information on individual medical history (diagnostic code, laboratory results from national health screening program, lifestyle factors, anthropometric data, and death information based on a death certificate) and socioeconomic data (based on insurance premiums and self-reported questionnaires). The data from the NHIS-HEALS cohort were based on real-world measurements in a clinical setting. The real-world data showed the true relationship reflecting many variables and environments that we did not expect. Second, an apparently healthy population aged 40 years or older who received a national health screening examination was enrolled in this study, while patients who were diagnosed with diabetes, cancers, or CCVDs were excluded from the analysis. In addition, to control the effects of medications, such as glucose-lowering and lipid-lowering drugs, individuals who had taken these drugs between 2002 and 2010 were excluded. However, individuals treated with these drugs after 2010 were not excluded in the final analyses because the aim of this study was to investigate the association between the initial TyG index and CCVDs or all-cause mortality regardless of medication during the follow-up period. In other words, we just tried to examine whether the elevated TyG index at baseline increases the CCVDs and all-cause mortality. These strict exclusion criteria may minimize the effects of underlying conditions on the primary outcome. Despite strict exclusion, many subjects (144,000 or more) were included in the final analyses. Third, the median follow-up duration was 5.97 years. In the case of apparently healthy individuals, large population size and longer study duration allowed a more accurate relationship to be explored than those from previous studies [27, 28]. Fourth, we widely examined the outcomes, which were related to the TyG index. The primary outcomes were stratified to several individual outcomes and sequentially investigated the relationships between them and the TyG index. In addition, many confounding factors including health behaviors, SBP, LDL-C, economic status, and anti-hypertensive medications for CCVDs were adjusted in the Cox proportional hazards regression models. This conservative approach provides a reliable association between the TyG index and outcomes to minimize the function of these conventional risk factors as confounders.

There are several limitations that should be interpreted cautiously. We could not validate whether the TyG index directly correlated with insulin resistance because the NHIS-HEALS cohort did not contain information on serum insulin levels. However, the TyG index was already validated to indicate insulin resistance in previous studies [14, 31]. The outcomes such as CVDs, CbVDs, and CCVD-related deaths were not directly collected by our research team. There is the possibility of misclassification or a different definition of outcome events. The Korean health insurance system is uniquely different from other countries because all Koreans should subscribe to the NHIS and the Korean government collects and controls health information, insurance claim information, and reimbursement. For example, the NHIS assesses the coverage of insurance and determines the cost of the individual medical service, while Health Insurance Review and Assessment Service (HIRAS), another national agency, reviews the claim data and reimburses the hospital fees. Special diseases such as CCVDs, cancers, and rare diseases are more strictly monitored by these two national agencies because patients with special diseases pay 5% of their hospital fee and the NHIS pays the remaining medical cost. These strict monitoring systems minimize inaccurate diagnosis. In addition, to minimize the misclassification, we more conservatively defined the CCVDs. CCVDs were defined when the relevant diagnostic codes (I20-I25 or I60-I69) as the main diagnostic code were recorded more than once at hospitalization or at least twice at the outpatient visit. Even if there are misclassifications, we believe that there would be very few. Repeatedly measured TyG index was not used in this study. The longitudinal study using repeated measurements of the TyG index was warranted to investigate the more accurate association between the TyG index and the outcomes such as CCVD and all-cause death than one measurement. The TyG index seems to be significantly affected by diet and ethnic group [11, 12]. Thus, the findings from the current study are difficult to generalize to all countries and ethnic groups. Menopausal status was not available in the NHIS-HEALS cohort. Menopause is a well-known risk factor of metabolic abnormality and cardiovascular diseases [32]. If we stratified women according to menopausal status, a more precise relationship could have been shown between TyG index and CCVD-related outcomes in women. Finally, newly prescribed glucose- and lipid-lowering drugs prescribed during the follow-up period was not adjusted in the Cox proportional hazards regression model. These drugs might affect the association between the TyG index and the outcomes.

In conclusion, men with an elevated TyG index were at higher risk for CCVDs or all-cause mortality. Individuals with a higher TyG index were more likely to develop CVDs and ischemic CbVDs in both sexes while the TyG index was not significantly associated with CCVD-related death and all-cause death.

## Supporting information

S1 Fig Kaplan-Meier estimates for cerebrovascular diseases according to triglycerides-glucose index quartile group. A. Ischemic cerebrovascular diseases. B. Hemorrhagic cerebrovascular diseases. C. Other cerebrovascular diseases. All p-values are from log-rank tests.
(TIF)

S1 Table Two-way ANOVA for the continuous variables and log-linear models with two-way interaction for the categorical variables by sex and TyG index quartile. (DOCX)

S2 Table Cox-proportional hazards regression model for the primary outcomes (cardio-cerebrovascular diseases or all-cause death) according to TyG index quartile. (DOCX)

## List of abbreviations

ANOVA: analysis of variance
BMI: body mass index
CbVD: cerebrovascular disease
CCVD: cardio-cerebrovascular disease
CI: confidence interval
CVD: cardiovascular disease
HEALS: health screening
HOMA-IR: homeostasis model assessment of insulin resistance
HR: hazard ratio
ICD: international classification of diseases
LDL-C: low-density lipoprotein cholesterol
NHIS: national health insurance service
SBP: systolic blood pressure
SD: standard deviation
TG: triglycerides
TyG: triglycerides-glucose
