# Supplementary figures and images for: The association between triglyceride-glucose index, cardio-cerebrovascular diseases, and death in Korean adults: A retrospective study based on the NHIS-HEALS cohort
Source: PLoS One. 2021 Nov 4;16(11):e0259212. doi: 10.1371/journal.pone.0259212 (PMC8568280; doi:10.1371/journal.pone.0259212)

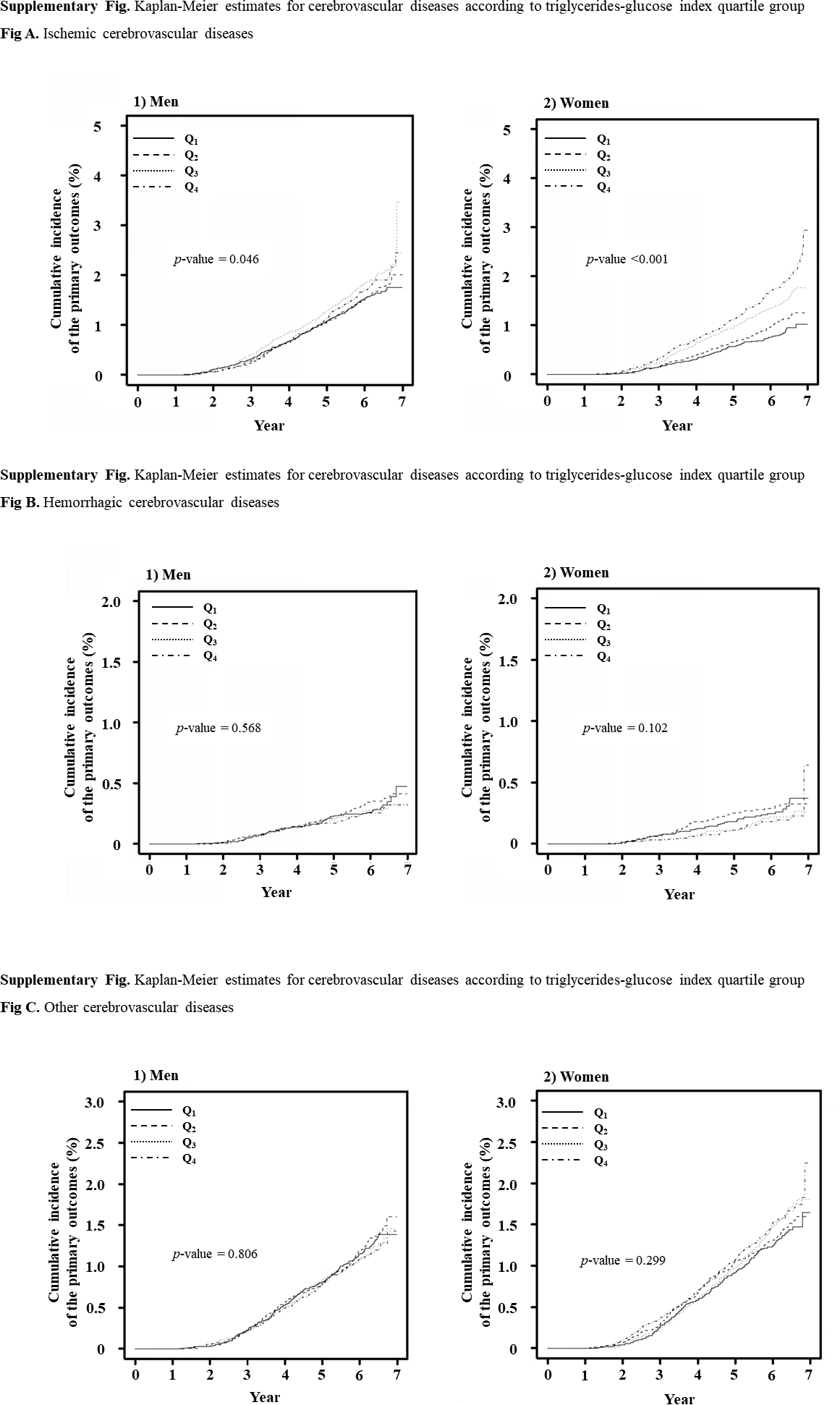

Supplement: S1 Fig — A. Ischemic cerebrovascular diseases. B. Hemorrhagic cerebrovascular diseases. C. Other cerebrovascular diseases. All p-values are from log-rank tests. (TIF) [file pone.0259212.s001.tif]
