# Supplementary material for: The association between triglyceride-glucose index, cardio-cerebrovascular diseases, and death in Korean adults: A retrospective study based on the NHIS-HEALS cohort
Source: PLoS One. 2021 Nov 4;16(11):e0259212. doi: 10.1371/journal.pone.0259212 (PMC8568280; doi:10.1371/journal.pone.0259212)
Supplement: S1 Table — (DOCX) [file pone.0259212.s002.docx]

**Supplementary Table 1.** Two-way ANOVA for the continuous variables and log-linear models with two-way interaction for the categorical variables by sex and TyG quartile

|  |  | Sex | TyG |
| --- | --- | --- | --- |
|  |  | p-value | p-value |
| Age |  | <0.001 | <0.001 |
| BMI, kg/m^2^ |  | <0.001 | <0.001 |
| SBP, mmHg |  | <0.001 | <0.001 |
| Glucose, mg/dL |  | <0.001 | <0.001 |
| TG, mg/dL |  | <0.001 | <0.001 |
| LDL-C, mg/dL |  | <0.001 | <0.001 |
| TyG |  | <0.001 | <0.001 |
| Ever smokers, N (%) |  | <0.001 | <0.001 |
| Drinking status, N (%) |  | <0.001 | <0.001 |
| Physical activity, N (%) |  | <0.001 | <0.001 |
| Economic status, N (%) |  | <0.001 | <0.001 |

Abbreviations: BMI, body mass index; TG, triglyceride; LDL-C, low-density lipoprotein cholesterol; TyG index, triglycerides-glucose index
