# Supplementary material for: The association between triglyceride-glucose index, cardio-cerebrovascular diseases, and death in Korean adults: A retrospective study based on the NHIS-HEALS cohort
Source: PLoS One. 2021 Nov 4;16(11):e0259212. doi: 10.1371/journal.pone.0259212 (PMC8568280; doi:10.1371/journal.pone.0259212)
Supplement: S2 Table — (DOCX) [file pone.0259212.s003.docx]

**Supplementary Table 2.** Cox-proportional hazards regression model for the primary outcomes (cardio-cerebrovascular diseases or all-cause deaths) according to TyG index quartile

| Models |  | Men | Women |
| --- | --- | --- | --- |
| Model 1 | Q_1_ | Reference | Reference |
|  | Q_2_ | 1.087 (1.005−1.177) | 1.123 (1.008−1.252) |
|  | Q_3_ | 1.155 (1.067−1.250) | 1.095 (0.984−1.219) |
|  | Q_4_ | 1.208 (1.115−1.310) | 1.139 (1.025−1.266) |
| Model 2 | Q_1_ | Reference | Reference |
|  | Q_2_ | 1.075 (0.993−1.163) | 1.121 (1.006−1.250) |
|  | Q_3_ | 1.131 (1.045−1.225) | 1.092 (0.981−1.216) |
|  | Q_4_ | 1.168 (1.077−1.267) | 1.134 (1.021−1.260) |
| Model 3 | Q_1_ | Reference | Reference |
|  | Q_2_ | 1.063 (0.982−1.152) | 1.099 (0.986−1.226) |
|  | Q_3_ | 1.112 (1.026−1.206) | 1.049 (0.941−1.169) |
|  | Q_4_ | 1.153 (1.060−1.254) | 1.069 (0.960−1.190) |
| Model 4 | Q_1_ | Reference | Reference |
|  | Q_2_ | 1.062 (0.981−1.150) | 1.099 (0.986−1.226) |
|  | Q_3_ | 1.110 (1.024−1.204) | 1.046 (0.938−1.166) |
|  | Q_4_ | 1.151 (1.058−1.252) | 1.063 (0.954−1.184) |

Model 1: adjusted for age

Model 2: adjusted for smoking status (ever and never smokers), drinking status (rare, sometimes, and often) and physical activity (rare, sometimes, and regular) in addition to the variable of Model 1

Model 3: adjusted for body mass index, systolic blood pressure, low-density lipoprotein cholesterol, and economic status (low, middle, and high), in addition to the variables of Model 2

Model 4: adjusted for anti-hypertensive medications, in addition to the variables of Model 3
